# Parents’ Creative Self-Concept and Creative Activity as Predictors of Family Lifestyle

**DOI:** 10.3390/ijerph17249558

**Published:** 2020-12-21

**Authors:** Izabela Lebuda, Dorota M. Jankowska, Maciej Karwowski

**Affiliations:** 1 Institute of Psychology, University of Wroclaw, 50-527 Wrocław, Poland; izalebuda@gmail.com (I.L.); maciej.karwowski@uwr.edu.pl (M.K.); 2 Institute of Education, The Maria Grzegorzewska University, 02-353 Warszawa, Poland

**Keywords:** creative activity, creative self-concept, family climate for creativity, family social functioning, family cohesion

## Abstract

Family rules, routines, and resources shape children’s creativity. However, little is known about how parents’ creative self-concepts and creative activity are related to the lifestyle adults create in their families. Family lifestyle might be operationalized as referring to domain-general dimensions of family social functioning (cohesion, flexibility, communication, and family satisfaction) and domain-specific factors related to creativity, namely, family climate for creativity (encouragement to experience novelty and varieties, encouragement to nonconformism, support of perseverance in creative efforts, encouragement to fantasize). To explore the link between parents’ creativity-related characteristics and family lifestyle, 303 Polish parents (57% mothers) of children aged between 6 and 10 (*M* = 7.99; *SD* = 1.38) reported on their creative self-concept (creative self-efficacy and creative personal identity), creative activity, overall family lifestyle, and climate for creativity in their families. We found that parents’ creative self-concept and their creative activity predict support for creativity in the family and more general balanced and satisfying family relationships. We discuss these findings, point new paths for future research, and suggest possible interventions to strengthen families as creativity-fostering environments.

## 1. Introduction

Everyday practice of parenting, as well as interpersonal relationships in the family, define the developmental context and set socialization goals [1]. Parental style and activities are related to children’s social skills [2], their psychological adjustment [3,4], as well as personal and social well-being [5,6]. Importantly, effective parenting might also serve as a protective factor for risk and unhealthy behaviors among young people [7]. Creativity literature, both classic [8,9,10,11,12,13] and contemporary [14,15,16,17,18,19,20], provides additional evidence of how parents influence children’s abilities and later achievements. While parental style and practices result from more general goals and values [1], still little is known about parents’ specific psychological characteristics and behaviors that might play a role in creating a family environment that supports development of abilities, beliefs, and attitudes conducive to children’s creativity. Supporting children’s creativity seems critical not only for economic and social reasons. Creativity was found to be the source of positive emotions [21,22,23,24], a way to self-fulfillment, self-actualization, and a characteristic of a healthy individual [25].

Although previous studies demonstrated that caregivers’ personality plays a role in explaining their parental styles, relationship with children, and children’s cognitive abilities [26,27,28,29,30], little is known about individual differences that relate to a family’s creative lifestyle [31,32]. We theorize that creative self-concept might relate to everyday family practice and intentional effort to support children’s creativity. Therefore, the main aim of the present research was to examine how creative self-concept, a factor responsible for agentic functioning [33], and parents’ creative activities, are related to the family lifestyle.

### 1.1. Parental Creativity-Related Characteristics

#### 1.1.1. Parents’ Creative Self-Concept

People’s goals, choices, and actions are driven by their beliefs [34]. In recent decades, it has been pointed out that creativity depends not only on creative abilities, but also on creative self-concept [33]. Creative self-concept describes people’s convictions about creativity [35]. Such convictions could be organized into three broad categories: creative confidence beliefs, creative self-awareness beliefs, and creative self-image beliefs [33]. Self-concept is often defined as a multidimensional construct depicting self-perceptions in specific domains [36] that consist of interrelated facets [37,38,39]. In the study we present below, we focused on two related aspects of creative self-concept: creative self-efficacy [40,41] and creative personal identity [42,43]. Creative self-efficacy is one of the creative confidence beliefs, a conviction that “one has the ability to produce creative outcomes” [41] (p. 1138) and refers to “a person’s perceived confidence to creatively perform a given task, in a specific context, at a particular level” [32] (p. 398). This belief is malleable, influenced by sociocognitive and environmental factors [34]. Creative confidence mediates the relationships between creative potential and creative behavior [44,45,46,47]. People who believe in their creative abilities are more engaged in creative tasks and challenges [41,44,47,48,49]. In light of existing studies, creative self-efficacy and creative personal identity are positively related, with creative self-efficacy being a more stable predictor of creative personal identity than the reverse [35,50,51].

While previous investigations demonstrated that eminent creators’ mothers were highly self-confident [52], we were unable to find any research about caregivers’ creative self-efficacy and their everyday behavior that may support children’s creativity. However, this link was quite intensively studied in the case of teachers [53]. Indeed, several studies have found that teachers with high self-efficacy and high creative self-efficacy are more prone to promoting students’ creativity and use teaching styles that support creativity [54,55,56]. Therefore, it could be assumed that parents who believe in their ability to deal with creative challenges will put more effort into creating a family lifestyle conducive to children’s creative development.

To act creatively, people not only have to be confident in their abilities, but also perceive creativity as important [57]. Creative self-image beliefs, especially creative personal identity, describe how someone values creativity and how significant this ability is for the sense of self [35,42,58]. Valuing creativity seems crucial for the decision to engage in creativity [59]. Creative actions help to achieve a congruent self-image as a person for whom creativity is an integral part of personal identity [60,61]. Valuing creativity moderates the relation between creative potential and creative behavior [58], so to employ creativity in any area of life, also in the everyday family context, people have to be confident in their abilities but also perceive creativity as valuable.

It is known that parents’ general self-image translates into parental styles and family climate [62]. In the case of parents’ creative self-image, we managed to find only a few premises about their role for a creative lifestyle. The first one is that parents with a positive attitude towards creativity and positive value of creative child characteristics are more prone to nurture children’s creativity [63]. An indirect link between creative self-image and family life was also presented in the qualitative analysis of eminent creators’ experience. It has been shown that beliefs about the importance of creativity in one’s life and perceiving the creator’s role are primarily related to how highly creative people fulfill family roles [64]. However, this research mainly focused on the role of being a spouse or a partner, less so on being a parent. Also, in qualitative analyses it was shown that mothers who valued creativity associated it mainly with a specific lifestyle and personal life and less so on professional creative activities. Their creative functioning tended to focus on two main areas: building a flexible, empathic relation with the child and creating a climate conducive to the child’s creative development [32].

#### 1.1.2. Parent’s Creative Activity

Creative activity refers to all levels of creativity, from generating ideas or preparing a new meal, through professional problem-solving, to socially recognized creative achievements, like publishing a book [65,66]. It was even pointed out that “the conduct of life in itself can be a creative act” [67] (p. 181). Everyday life actions, relations with others, and dealing with daily duties and unexpected challenges often need creativity. This kind of activity, usually called everyday creativity [68,69], refers to non-eminent, nonprofessional behaviors defined in terms of “human originality at work and leisure across the diverse activities of everyday life” [70] (p. 190). In this perspective, arranging family life and supporting children’s development could be considered a creative activity [32]. Parenthood, besides mating and work, is one of the primary forms of an adult person’s creative form of expression [71]. Parents agree that their children are a source of inspiration and that parenthood makes them more creative [72]. In the case of people for whom creativity is an essential part of their identity, parenthood lets them fulfill their creative needs and helps to manage their family duties, work, and personal life, as well as to deal effectively with various demands associated with different social roles [32]. Therefore, parents’ creativity is mostly linked to the efficiency of solving problems and challenges in an unusual way [32].

Classic studies have found that mothers of highly creative children were often deeply involved in their careers and had active and independent life [73]. At the same time, mothers of more creative children felt more self-fulfilled at home and more accomplished at work than mothers of less creative ones [10]. The different relations between parenthood and creative activities is illustrated by a typology derived from biographies of acclaimed female artists [74]. Five types of relationships were pointed out: (1) give up parenthood in favor of creative work; (2) be a parent, but focus on own creative development, limit taking care of a child to a minimum; (3) focus mainly on parenthood and limit or withdraw from creative activities at all; (4) try to balance parenting with outside family creative activities; (5) engage in parenthood and other creative activities sequentially [74]. It might be expected that parents who are more creative in everyday life will also establish a more creative family environment. Still, however, it is equally possible that if they engage in family life, they will abandon outside-family creative activities.

### 1.2. The Creative Lifestyle

We define the creative lifestyle as a family’s everyday practice and interpersonal relationships, especially communication tendencies, which may directly and indirectly support creative development of all family members. Two main areas that constitute the creative lifestyle are (1) domain-general, such as social functioning of the family, and (2) domain-specific, such as the climate for creativity in the parent–child relationship.

#### 1.2.1. Family Social Functioning

Apart from building the family climate, making relationships with children is an essential creative parental activity [32]. One of the most popular theories and assessment of family social performance [75] is reflected in the Circumplex Model of Marital and Family Systems, which contains six dimensions: cohesion, flexibility, disengaged, enmeshed, rigid, and chaotic [76]. In opposition to parenting style, in which such dimensions as responsiveness and demandingness are traditionally conceptualized as orthogonal constructs [77,78,79], the Olson’s model consists of related aspects. Many studies confirmed that cohesion and flexibility were highly and positively correlated and negatively correlated with the unbalanced scales, mainly with disengagement and chaos e.g., [80,81]. There are two main dimensions: cohesion and flexibility. Cohesion (togetherness) describes the emotional bonding between family members [81]. Emotional closeness, time spent together, a common form of relaxation, and decision-making consulted with each other are some indicators of cohesion in a family. The spectrum of cohesion is from enmeshed, through connected, separated, to disengaged. Flexibility describes how stability is balanced with a change in a family [81]. The spectrum of flexibility ranges from chaotic, through flexible, structured, to rigid. Some indicators of flexibility in a family are that all family members participate in making rules and the entire family is looking for new ways of dealing with challenges. Main hypotheses derived from the Circumplex Model are that balanced families are more functional, have more positive communication, and more effectively modify their cohesion and flexibility to deal with changes and challenges. Cohesive families balance between separateness and togetherness—family members are both independent and connected to family. High cohesion means too little independence in family; too low means limited attachment to it. High level of flexibility could lead to chaos, and low flexibility could lead to rigidity. A balanced family is structured and flexible at the same time.

Communication and family satisfaction are two additional factors of the model [81]. Communication helps alter the appropriate level of cohesion, and flexibility allows us to deal with challenges, facilitate, and maintain a balanced relationship. Family satisfaction indicates how much each family member likes the current family system.

A few previous studies investigated the links between the Circumplex Model of family functioning and children’s creative potential. It was observed that freshman college students from highly adaptable families obtained the highest self-reported creativity and the lowest scores were noted in the case of ones from rigid families (low adaptability) [82]. Family cohesion was not particularly important for creativity. The authors speculated that while growing up in adaptable families (flexible and even chaotic), people can make their own mistakes, learn from them, and develop creativity [82].

The results of a study on preschool children’s creative potential and family social functioning demonstrated that children from more flexible families had slightly higher scores on originality, while children from more cohesive (enmeshed) families were less likely to score high on creativity measures [83]. However, high family cohesion was also a positive predictor of perceived own creativity among gifted Chinese students [84]. The difference in results could be a matter of cultural difference, but it could also be derived from different measures applied. It also cannot be ruled out that there are two paths facilitating the development of creativity [85]—one associated with freedom and rejection (called distance-conflicted family theory; see e.g., [86]) and the other with warmth, support, and acceptance (called autonomy-supportive family theory; see e.g., [9]).

#### 1.2.2. Family Climate for Creativity

Climate for creativity in the parent–child relationship is defined as “parents’ overall relatively constant behavioral pattern that helps the child acquire a mindset, attitudes, personal qualities, and skills necessary for creativity” [31] (p. 14). It is a stable pattern of parents’ attitudes and behaviors toward children aimed at enhancing creativity [87]. Four key factors of climate for creativity in a parent–child relationship have been identified to-date: encouraging the pursuit of novel and varied experience, supporting a nonconformist attitude and independence, strengthening perseverance in the performance of creative tasks, and encouraging and supporting fantasizing [31,32]. They all are instrumental in strengthening children’s overall development, with a particular focus on creativity development. Gaining novel and diversified experiences constitutes a form of the cultural capital of a child that enhances creativity by stimulating new ideas and interests [15,52]. Independence and nonconformism were important predictors of creative activity and achievements [88]. The same applies to perseverance—crucial for creative achievement [89], with a unique role played by parents in modeling this kind of behavior [15]. The last factor is active parental support of children’s fantasizing. Indeed, it has been demonstrated that creative imagination is one of the crucial creative abilities [90,91,92], and play combined with the fantasy is one of the predictors of adult creativity [92].

It has been shown that the Big Five personality traits predict the climate for creativity in mothers’ relationships with children [31]. All four creative climate dimensions were predicted by mothers’ openness to experience. Additionally, encouragement for children to seek novelty and variety was positively related to mothers’ emotional stability, conscientiousness, and extraversion, while supporting a nonconformist attitude and independence was negatively predicted by conscientiousness. Strengthening perseverance in the performance of creative tasks was linked to mothers’ emotional stability, conscientiousness, agreeableness, and extraversion.

Other studies have found that personality and creative self-concept are related yet separate constructs [93,94]. Thus, we decided to examine how parents’ creative self-efficacy and their creative personal identity link with four factors of family climate for creativity. Due to the fact that it was shown that aspects of the family climate for creativity relate with the constructive parenting style—parental acceptance and autonomy granting [95]—we also expected that they would be connected with family creative lifestyle. Accordingly, we hypothesized that parents’ creative self-concept and creative activity would be positively related to the family’s social functioning and the family climate for creativity.

The purpose of this study was to examine how parent-related factors (parents’ creative self-concept and creative activity) are linked to family lifestyle. Family lifestyle refers to essential dimensions of family functioning (cohesion, flexibility, communication, family satisfaction) and the climate for creativity in a parent–child relationship (encouragement to experience novelty and varieties, encouragement to nonconformism, support of perseverance in creative efforts, encouragement to fantasize). We hypothesized that parents’ creativity—both creative self-concept and creative activity—would be positively related to the family’s social functioning and the family’s climate for creativity.

## 2. Materials and Methods

### 2.1. Participants

In the analysis, we included data from 303 Polish parents (41% biological fathers, 57% biological mothers, 2% others—legal guardians of the child) of children between 6 and 10 years of age (*M* = 7.99; *SD* = 1.38). Mothers’ ages ranged from 23 to 53 (*M* = 37.32, *SD* = 5.77, fathers’ from 27 to 64 (*M* = 40.10; *SD* = 6.52).

The survey participants came from Poland, from cities with: (a) population over 100,000 (37%), (b) population between 20 and 100,000 (24%), (c) population under 20,000 (11%), and (d) lived in rural areas (28%) (variable controlled at sampling level). In our study, we controlled sociodemographic variables, such as parent’s gender, age, level of education, self-estimation of material situation, and the number of children. The parents/guardians were asked through a questionnaire to indicate separately for the father and the mother the highest level of their education (elementary, secondary, bachelor’s degree, master’s degree, doctorate) and assess on a five-point scale the material situation of the family (1 = only for the basic needs, 5 = we have earnings). Information about gender, age, and number of children was gathered in open-ended questions.

We estimated the required sample size in GPower 3.1.96 [96] for regression analysis based on three assumptions. First, we expected that the effect size of the links would fall between weak and moderate (f^2^ = 0.11), so that parents’ creativity (creative self-concept and creative activity) and control variables would explain about 10% of the family’s social functioning and creative climate variance. Second, we estimated the required sample size for regression models with eight predictors: three main variables (creative self-efficacy, creative personal identity, creative activity) and five control variables (parents’ sex, age, level of education, family’s material situation, and a number of children—see description below). Third, and finally, given that we planned a series of independent regression analyses (eight for family’s social functioning and four for family’s climate for creativity), we corrected the critical alpha level to 0.05/12 = 0.004. The beta level was set to 0.90. The required sample size to find a small-to-moderate effect with these assumptions was estimated at *N* = 270. Given that family lifestyle might be considered a variable that is socially embedded, we considered it important to control for several confounds, starting from parents’ age and sex, to their education and material situation.

### 2.2. Measures

All variables, sample items, and scales’ reliabilities are described in detail below and summarized in Table 1.

#### 2.2.1. Parents’ Creative Self-Concept

The Short Scale of Creative Self [51] was used to measure parents’ creative self-concept. The instrument consists of eleven items. Six items measure creative self-efficacy (example: I know I can efficiently solve even complicated problems, *α* = 0.92). Five items are dedicated to assessing creative personal identity (example: My creativity is important to who I am, *α* = 0.91). A 7-point scale was used: 1 = definitely not, 7 = definitely yes.

#### 2.2.2. Parents’ Creative Activity

A modified version of the Inventory of Creative Activities and Achievements [88,97] (activity scale only) was used to measure parents’ creative activity. The instrument includes 33 items with creative activities in seven domains: (a) everyday (7 items), (b) web development/programming (7 items), (c) music (4 items), (d) science (4 items), (e) dance (2 items), (f) visual arts (4 items), and (g) writing (5 items). Participants reported whether they engaged in a particular activity within the last year, from 0 = never to 4 = more than ten times (*α* = 0.91).

#### 2.2.3. Family Climate for Creativity

Family climate for creativity was measured using the Climate for Creativity in Parent–Child Relationship questionnaire. It is a 24-item instrument, measuring four dimensions of the climate for creativity in the parent–child relationship: Encouragement to Experience Novelty and Variety (example: I try to suggest to my child unconventional ways to solve problems, *α* = 0.84), Encouragement of Nonconformism (example: I do not want my child to stand out from the group (*α* = 0.83), Support of Perseverance in Creative Efforts (example: When my child has problems I support and motivate him/her to see many solutions, *α* = 0.93), and Encouragement to Fantasize (example: I sometimes engage my child in my “weird” ideas, *α* = 0.80) [31,32,98]. Each scale consists of six items, each using a seven-point response scale (1 = entirely disagree; 7 = entirely agree).

#### 2.2.4. Family Social Functioning

Family Social Functioning was measured by a Polish adaptation [99] of FACES IV Flexibility and Cohesion Evaluation Scales. This 62-item instrument consists of six basic scales (7 items each); two balanced: balanced cohesion (example: Family members are involved in each other’s lives, *α* = 0.78), balanced flexibility (example: Our family tries new ways of dealing with problems, *α* = 0.70); 4 unbalanced: disengaged (example: We get along better with people outside our family than inside, *α* = 0.81), enmeshed (example: We spend too much time together, *α* = 0.76), rigid (example: There are strict consequences for breaking the rules in our family, *α* = 0.63), and chaotic (example: We never seem to get organized in our family, *α* = 0.77). Two additional scales (10 items each) are family communication (example: Family members are satisfied with how they communicate with each other, *α* = 0.94) and family satisfaction (The degree of closeness between family members, *α* = 0.92). Responses are provided on a 5-point Likert scale (from 1 = I completely agree, to 5 = I completely disagree).

#### 2.2.5. Control Variables

We controlled for parents’ gender, age, education level (elementary, secondary, bachelor’s degree, master’s degree, doctorate), self-assessed material situation (1 = poor, 5 = very good, *M* = 2.45; *SD* = 1.09) as well as the number of children (min = 1, max = 5, *M* = 2.03; *SD* = 0.93). Information about gender, age, and number of children was gathered in open-ended questions.

### 2.3. Procedure

The study was conducted online using the Pollster company online research panel. Data collection took about 2 weeks. One parent per family contributed data; all participants had one or more children between 6 and 10 years of age. Participants were informed about confidentiality of their responses and provided consent to process personal data. The protocol and procedure of the study were accepted by the Institutional Review Board of the 2nd author’s university. Participants were required to answer all questions to follow the survey, so there were no missing values. The data were collected in 2020.

### 2.4. Data Analysis

For the purpose of this study, we decided to integrate variable- and person-centered analyses. To quantify the amount of variability of family social functioning and family climate for creativity associated with parents’ individual characteristics (sex, age, education), family situation (number of children and material condition of the family), as well as their creativity (creative self-efficacy, creative personal identity, and creative activity), we relied on regression analysis. More specifically, in subsequent models, we regressed family lifestyle variables on creativity-relevant characteristics and controls. Regarding the person-centered analyses, we conducted a number of latent profile analyses in MPlus 8.1, with family social functioning and family climate for creativity as criteria of differentiation of the latent profiles. We tested three solutions consisting of 2, 3, and 4 latent profiles. In deciding which solution should be saved for further analyses, we used statistical criteria (sample size-adjusted Bayesian Information Criteria (SSA BIC), Lo–Mendell–Rubin Likelihood Ratio Test (LRT), and entropy) as well as profiles’ interpretability. Finally, to estimate potential differences between profiles, we relied on analyses of variance (ANOVAs), with creative self-efficacy, creative personal identity, and creative activity as dependent variables and profiles as factors.

## 3. Results

Descriptive statistics and sample items are presented in Table 1. Correlations between variables used in this study are presented in the Appendix A Table A1. Some associations between variables were unexpected. In family social functioning, it was the positive link between balanced flexibility and rigidity (*r* = 0.29; *p* < 0.05). In the case of family climate for creativity, contrary to our expectations, some scales linked negatively to each other; for instance, encouragement of nonconformity was negatively linked with encouragement of novelty (*r* = −0.53; *p* < 0.05), support of perseverance (*r* = −0.70; *p* < 0.05), and encouragement of fantasy (*r* = −0.47; *p* < 0.05). Also, two correlations between family functioning and creativity encouragement were opposite to what could be expected based on literature: a negative link between balanced flexibility of family and encouragement of nonconformity was observed (*r* = −14; *p* < 0.05), similarly to the negative link between communication and encouragement of novelty (*r* = −12; *p* < 0.05).

Separate models were created for each of the family social functioning scales (See Table 2) and family creative climate scales (See Table 3).

### 3.1. Parents’ Creativity and Family Social Functioning

As illustrated in Table 2, controls were generally weakly associated with family lifestyle variables. Fathers tended to be more disengaged, enmeshed, rigid, and chaotic than mothers. Higher rigidity and chaos were also typical for younger parents. Family satisfaction was positively linked with the self-reported material situation of the family.

Creative self-efficacy was a consistent predictor of positive aspects of family lifestyle (βs from 0.28 in the case of balanced cohesion to 0.40 in the case of family satisfaction) and was negatively linked to all negative aspects of family lifestyle except for rigidity (βs from −0.19 in the case of feeling enmeshed to −0.26 in the case of the assessed chaotic situation in the family). The two remaining creativity-related variables were less consistent in predicting the family lifestyle. Creative personal identity positively predicted balanced cohesion, communication, and family satisfaction, while it negatively predicted a chaotic lifestyle. Creative activity was unrelated to the family lifestyle.

### 3.2. Parents’ Creativity and Family Climate for Creativity

A model estimated for creative climate factors yielded similar results. Controls were generally unrelated to creative climate characteristics, apart from the educational level, which positively predicted encouragement of novelty. Similarly to the previous case, creative self-efficacy was positively associated with encouragement of novelty, fantasy, and supporting perseverance, while being negatively linked with encouragement nonconformity. Creative personal identity explained a unique portion of the variability in encouragement of novelty and supporting perseverance while being independent of encouraging nonconformity and encouraging fantasy. Creative activity was positively linked only to encouraging fantasy.

### 3.3. Latent Profile Analysis

Our variable-centered analyses provided a consistent yet not very compelling pattern. Although creativity-related variables tended to be positively associated with family social functioning and family climate for creativity, the effect size of these links was generally weak-to-moderate, only rarely (family satisfaction) explaining the robust portion of its variability. However, given that family factors tend to cluster and form a more complex pattern of styles, we decided to provide an additional set of analyses—this time person-centered. As demonstrated in Table 4, the 2-profile solution was significantly better than the 1-profile solution, as illustrated by a significant LRT test. The three-profile solution was characterized by an even better fit, as represented by a lower SSA BIC and significant value of LRT. The four-profiles solution held lower SSA BIC than the 3-profiles solution, yet the LRT test has shown that its fit was not significantly better than in the case of three profiles. Therefore, we decided to proceed with a three-profile solution.

**Figure 1:**
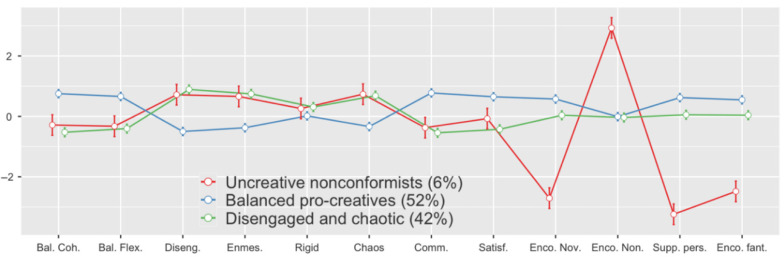
Differences in criteria factors used in the Latent Profile Analysis.

As illustrated, the three profiles had clear and distinctive characteristics (Figure 1). The smallest, Profile 1 (6%), was characterized by very high encouragement of nonconformity in parent–child relations, yet this nonconformity was accompanied by very low support for other factors associated with creativity. Therefore, we decided to call this profile “uncreative nonconformists.” The two remaining profiles were large: the largest one (52%) was composed of parents who assessed their families as balanced, were satisfied, and supported creativity of their children. We called this profile “balanced pro-creatives.” The last profile (42%)—in a sense—reversely mirrored Profile 2. These parents tended to be disengaged, enmeshed, poor in communicating, and not satisfied with their family life. Their support for the creativity of their children was low or moderate. We called them “disengaged and chaotic”.

In the case of creative self-efficacy, we observed significant differences with a moderate effect size, *F*(2, 300) = 23.53, *p* < 0.001, ω^2^ = 0.13. A post hoc test with Tukey correction for multiple comparisons demonstrated that there was significant difference between “balanced pro-creatives” (*M* = 5.38, *SD* = 0.91) and “disengaged and chaotic” (*M* = 4.61, *SD* = 1.03), *M*_diff_ = 0.76, *SE* = 0.11, *p* < 0.001, Cohen’s *d* = 0.80. We emphasize the large effect of these differences. “Balanced pro-creatives” also scored higher than “uncreative nonconformists” (*M* = 4.87, *SD* = 0.74), yet this difference did not survive Tukey correction (*M*_diff_ = −0.51, *SE* = 0.23, *p* = 0.079, Cohen’s *d* = −0.57). There were no differences between the profiles of “disengaged and chaotic” and “uncreative nonconformists” (*M*_diff_ = 0.26, *SE* = 0.24, *p* = 0.52, Cohen’s *d* = 0.26.

In the case of creative personal identity, ANOVA demonstrated a significant omnibus effect with robust effect size, *F*(2, 300) = 18.57, *p* < 0.001, ω ^2^ = 0.10. Similarly as in the previous case, there was a statistically significant difference between “balanced pro-creatives” (*M* = 5.32, *SD* = 0.96) and “disengaged and chaotic” (*M* = 4.57, *SD* = 1.18), *M*_diff_ = 0.75, *SE* = 0.12, *p* < 0.001, Cohen’s *d* = 0.71. “Balanced pro-creatives” tended to score higher than “uncreative nonconformists” (*M* = 4.80, *SD* = 0.67), yet this difference did not survive Tukey correction, *M*_diff_ = −0.53, *SE* = 0.26, *p* = 0.11, Cohen’s *d* = −0.56). There were no differences between “disengaged and chaotic” and “uncreative nonconformists” (*M*_diff_ = 0.22, *SE* = 0.26, *p* = 0.67, Cohen’s *d* = 0.20).

In the case of creative activity, the overall effect was significant, yet weaker in terms of the effect size, *F*(2, 300) = 7.76, *p* < 0.001, ω ^2^ = 0.043. “Balanced pro-creatives” (*M* = 1.69, *SD* = 0.46) reported significantly higher creative activities than “disengaged and chaotic” (*M* = 1.47, *SD* = 0.47), *M*_diff_ = 0.22, *SE* = 0.06, *p* < 0.001, Cohen’s *d* = 0.48, but they did not differ from “uncreative nonconformists” (*M* = 1.66, *SE* = 0.67), *M*_diff_ = −0.03, *SE* = 0.12, *p* = 0.96, Cohen’s *d* = −0.07. “Disengaged and chaotic” and “uncreative nonconformists” did not differ in terms of creative activity either (*M*_diff_ = 0.19, *SE* = 0.12, *p* = 0.26, Cohen’s *d* = 0.38).

## 4. Discussion

In this study, we sought to understand better whether, and if so then which, parents’ creativity-related characteristics (i.e., creative self-concept and their creative activity) are related to family lifestyle, considered as family social functioning (cohesion, flexibility, communication, and family satisfaction) and family climate for creativity (encouragement to experience novelty and varieties, encouragement to nonconformism, support of perseverance in creative efforts, encouragement to fantasize). As far as we are aware, our study was the first to test how parents’ creativity predicts such a comprehensively operationalized family lifestyle. Previous studies focused on one of these specific areas, namely either on the social context of family functioning [82,83,84], or on the climate for creativity built in the home environment [14,32]. Moreover, the relationship between parents’ creative self-efficacy and their behaviors that may support their offspring’s creativity has not yet been analyzed, while its importance seems natural.

The results of this might be summarized in three key findings. Firstly, creativity-related characteristics of parents are positively associated with positive aspects of family creative lifestyle. Secondly, creative self-concept of parents, especially their creative self-efficacy, predicts both domain-general dimensions of positive family and domain-specific factors related to creativity. Finally, there are latent profiles of parents that have distinctive characteristics and systematically differ from one another in terms of creative self-efficacy, creative personal identity, and creative activity.

Creative self-efficacy was a consistent predictor of balanced cohesion, flexibility, family satisfaction, and communication. At the same time, it was negatively linked to the family’s problematic functioning (i.e., disengagement, feeling enmeshed and chaotic situation in the family). Creative personal identity positively predicted balanced cohesion, communication, and family satisfaction while being negatively related to a chaotic lifestyle. We expected that parents’ creative self-concept and real-life creative activity would be more important for family lifestyle measured in a specific domain (i.e., for the family climate for creativity). Contrary to predictions, parents’ actual creative activity was unrelated to the family’s lifestyle, even when family lifestyle was assessed in domain-specific factors related to creativity.

Creative activity turned out to be positively related, but not very strongly, only to encouraging children to use fantasy. There are two possible explanations for this result. First, it seems most likely that parents’ creative activity may be related to family lifestyle, provided that this activity is undertaken with other family members, including children (for example, creative leisure activities [100]). Overall, leisure experiences play a vital role in family lifestyle because they build social relationships, positive emotions, cognitive stimulation, self-expression, and creativity [101,102]. Secondly, this effect may be due to the age of the children whose parents participated in our study. Imaginative play of preschool-age and early school-age children is less dependent on props than in previous developmental stages; it becomes more abstract and creative [103]. Maybe this is a clear symptom of children’s creativity for parents who undertake creative activities themselves and therefore make efforts to support creative fantasizing in play.

To better understand the relationships between parents’ creativity-related factors and family lifestyle dimensions, we used a person-centered approach—a latent profile analysis. Three profiles that emerged were named: “uncreative nonconformists,” “balanced pro-creatives,” and “disengaged and chaotic.” Each of these profiles showed a different pattern of correlates with family lifestyle variables. Parents who formed the “balanced pro-creatives” profile assessed their family as balanced, enjoyed higher satisfaction with their family life, and declared support for the offspring’s creativity. In contrast, a slightly smaller proportion of the “disengaged and chaotic” profiles reported a more problematic family system (enmeshed or disengaged relationships, poor family communication, and dissatisfaction with family relationships) and declared support for children’s creativity, but to a lesser extent. Juxtaposing of these two profiles suggests that both a balanced relationship with one’s own family and disharmony within the family system [76] might be associated with a family creativity-fostering environment [9,12,31]. Thus, it suggests two distinct paths to fostering creativity and building a creative lifestyle within the familial context. This finding corresponds with previous research, which on the one hand showed that families that provide warmth, support, and acceptance (probably a well-functioning family environment) are associated with children’s creativity and creative achievements in one’s later life [9,15], yet on the other that creative people often come from families that are anything but harmonious [84,104]. A recent study also provided empirical support for these two seemingly contradictory family theories in predicting creativity (i.e., the supportive-autonomy parental theory and distance-conflict parental theory [85]).

The identified profiles were characterized by statistically significant and theoretically meaningful differences in creative self-efficacy, creative personal identity, and declared creative activity. In the case of creative self-concept, parents classified into the “balanced pro-creatives” profile held substantially higher creative self-efficacy and creative personal identity than parents from the “disengaged and chaotic” profile. There were no differences between the profiles of “disengaged and chaotic” and “uncreative nonconformists,” or “uncreative nonconformists” and “balanced pro-creatives.” Parents’ creative activity formed the other creativity-related facet that displayed statistically significant differences across the latent profiles in this study. Representatives of the “balanced pro-creatives” group reported higher engagement in creative activities than the “disengaged and chaotic” ones. Similarly as in the previous case, we found no other statistically significant differences between the identified profiles in terms of the declared creative activity. These results partially confirm that parents with a positive attitude towards creativity are more likely to nurture their children’s creativity [63]. In that sense, the highest scores for all creativity-related variables seen in the “balanced pro-creatives” profile showed a similar pattern of relationships as in research with teachers [54,55,56], which we mentioned in the introduction. This similarity only partially explains our findings, but it does indicate that if parents believe in their ability to deal with creative challenges, and creativity is essential to them, they put more effort into their children’s creative development.

## 5. Limitations and Future Research

This investigation suffers from a few limitations that should be addressed in future studies. The first one is the character of the sample; all participants were from one country—Poland. Although in most of the psychological studies, the samples have come from one of the WEIRD (Western, educated, industrialized, rich, and democratic) countries [105], and in the creativity research the results from Poland were successfully compared with the results from more diverse samples (see for example [106]), it is worth being cautious of this limitation before generalizing the results of the research.

The second limitation is that all measures used in our study are self-reported instruments. We emphasize that all main variables we were interested in are traditionally investigated using questionnaires. However, it is worth considering that this type of assessment is prone to some problems, such as social desirability bias or common method variance. In the case of measuring the climate for creativity in the family, future research should specifically look at the scale of support for nonconformism. This issue was already raised elsewhere (see [95]). Our tentative explanation of the unexpected pattern of links observed in the present study considers children’s age as the potential reason. The parents of such young children may not define nonconformism as independence, courage, or high self-esteem, but as a rebellion that hinders effective functioning in society, and for this reason, they answered questions about this area in socially desirable ways. Therefore, this issue requires further clarification. The positive correlation between balanced flexibility and rigidity in our study (*r* = 0.29, *p* < 0.05) is even more surprising. Future studies would benefit from using more dynamic methods (e.g., observation of parent–child dyad across different contexts or tasks) to examine flexibility and rigidity in parent–child interactions (see [107]).

The third limitation is that we obtained all information only from the parents’ perspective. Future research should include other informants, such as children or outside observers. Including children in the study would allow them to compare their views with parents’ judgments and assess the relationship between parents’ self-concept, the family environment they arrange, and their children’s abilities, beliefs, and creative behaviors. While measuring children’s creativity is difficult or impossible for some reason, mothers’ ratings might be used to assess the creative performance of children (see Creative Activities Check List; [108]). As we did not examine children’s creativity in this study, we can only assume, based on previous research, that chosen aspects of family life support children’s creativity development. Future longitudinal studies could help understand how parental creativity-related characteristics and family environment influence changes in children’s abilities and beliefs crucial for creative potential realization.

Finally, we note that we utilized an instrument originally designed to measure domain-general creative self-efficacy and creative personal identity. However, given that creative self-concept is dynamic and task-related [35], future research would benefit from measuring how people value creativity in their parenting roles and how they judge their ability to deal with parenting challenges creatively. Moreover, we encourage future researchers to employ more than one type of creativity assessment to uncover a more comprehensive picture of parental creativity.

## 6. Conclusions

We found that parents’ characteristics predict creative family lifestyle—parents’ creative self-concept (creative self-efficacy and creative personal identity) and creative activity are associated with balanced and satisfying family relationships and with support for children creativity. Moreover, in the latent profile analysis, three distinct profiles of parents emerged: “uncreative nonconformists,” “balanced pro-creatives,” and “disengaged and chaotic.” Both parents from the “balanced pro-creatives” profile and the “disengaged and chaotic” profile reported support for children’s creativity, albeit to a different extent. It suggests that family creativity-fostering environment could be both balanced (in line with the autonomy-supportive family theory) and disharmonious (in congruence with distance-conflicted family theory).

## 7. Implications

As creativity is the source of positive experience in life [21,22,23,24,25], it seems worth it to try to better understand a family environment that supports development of abilities, beliefs, and attitudes conducive to children’s creativity. The creative family lifestyle, as we conceptualize it in the manuscript, should be subject to further investigations. This knowledge might be used to develop effective intervention programs. Moreover, future studies could examine interventions to help facilitate parents’ creative characteristics to enhance creative family lifestyle. Especially promising could be attempts at developing parent creative self-efficacy and creative self-identity, as creative self-concepts are considered malleable and changeable under external influences [33,34].

## Figures and Tables

**Table 1:** A Summary of variables included in the study and their descriptive statistics.

Variables	Sample Item	*M*	*SD*	Reliability
Creativity Variables (Parents’ Creative Self-Concept and Creative Activity)				
Creative Self-Efficacy	I know I can efficiently solve even complicated problems	5.05	1.03	0.92
Creative Personal Identity	My creativity is important to who I am	4.99	1.11	0.91
Creative Activity	I created an original decoration	1.60	0.49	0.91
Family Social Functioning				
Balanced Cohesion	Family members are involved in each other’s lives	4.07	0.61	0.78
Balanced Flexibility	Our family tries new ways of dealing with problems	3.75	0.59	0.70
Disengagement	We get along better with people outside our family than inside	2.15	0.79	0.81
Enmeshment	We spend too much time together	2.35	0.75	0.76
Rigid	There are strict consequences for breaking the rules in our family	2.90	0.61	0.63
Chaotic	We never seem to get organized in our family	2.52	0.75	0.77
Communication	Family members are satisfied with how they communicate with each other	3.93	0.76	0.94
Family satisfaction	The degree of closeness between family members	3.63	0.64	0.92
Family Climate for Creativity				
Encouraging Novelty	I try to suggest to my child unconventional ways to solve problems	5.23	1.11	0.84
Encouraging Nonconformity	I do not want my child to stand out from the group [reversed scored]	2.84	1.09	0.83
Supporting Perseverance	When my child has problems I support and motivate him/her to see many solutions	5.82	1.16	0.93
Encouragement Fantasy	I sometimes engage my child in my “weird” ideas	5.11	1.08	0.80

**Table 2:** Results of hierarchical regression analysis, with family social functioning variables as dependent variables. Presented are standardized coefficients (β).

Variables	Bal. Coh.	Bal. Flex.	Disengag.	Enmes.	Rigid	Chaotic	Communic.	Family Sat.
Sex	0.08	−0.01	0.21 ***	0.29 ***	0.17 **	0.18 **	−0.05	0.01
Age	0.03	0.05	−0.13 *	−0.07	−0.14 *	−0.23 ***	0.14 *	0.02
Education	0.03	−0.02	−0.07	−0.08	−0.03	0.04	−0.01	0.01
Material	0.01	−0.02	0.01	0.02	−0.03	0.02	−0.05	0.01
No. of Child.	−0.02	0.05	0.05	−0.00	0.15 *	0.04	−0.00	0.03
Creative Self-Efficacy	0.28 ***	0.38 ***	−0.23 ***	−0.19 ***	0.03	−0.26 ***	0.34 ***	0.40 ***
Creative Personal Identity	0.15 **	0.11	−0.05	0.03	0.00	−0.14 *	0.21 ***	0.31 ***
Creative Activity	0.08	0.04	0.03	0.07	0.10	0.03	0.08	0.02
R^2^	0.13	0.16	0.09	0.11	0.05	0.12	0.19	0.27

Note. *N* = 303. Sex coded 1 = woman, 2 = man; Bal. Coh. = balanced cohesion; Bal. Flex. = balanced flexibility; Diseng. = disengagement; Enmes. = enmeshed; Rigid. = rigidity; Chaotic = chaos; Communic. = communication; Family. Sat = family satisfaction. To avoid multicollinearity, creative self-efficacy and creative personal identity were introduced to the model as varimax-rotated factor analysis results. * *p* < 0.05, ** *p* < 0.01, *** *p* < 0.001.

**Table 3:** Results of hierarchical regression analysis, with family climate for creativity variables as dependent variables. Presented are standardized coefficients (β).

Variables	Enc. Nov.	Enc. Nonc.	Supp. Persev.	Enc. Fantas.
Sex	−0.03	−0.02	−0.10	−0.06
Age	0.01	0.02	0.02	−0.01
Education	0.12 *	0.03	0.03	0.04
Material	−0.08	0.14 *	−0.10	−0.08
No. of Child.	0.00	−0.05	0.01	0.02
Creative Self-Efficacy	0.28 ***	−0.14 *	0.26 ***	0.21 ***
Creative Personal Identity	0.14 *	−0.06	0.13 *	0.11
Creative Activity	0.12	0.09	0.01	0.16 **
R^2^	0.15	0.04	0.09	0.11

Note. *N* = 303. Sex coded 1 = woman, 2 = man; Enc. Nov. = encouragement novelty; Enc. Nonc. = encouragement nonconformity; Supp. Persev. = support for perseverance; Enc. Fantas. = encouragement fantasy. To avoid multicollinearity, creative self-efficacy and creative personal identity were introduced to the model as varimax-rotated factor analysis results. * *p* < 0.05, ** *p* < 0.01, *** *p* < 0.001.

**Table 4:** Comparison of the model fit with 2, 3, and 4 latent profiles in latent profile analysis.

Solution	SSA BIC	LRT Test	Entropy
2 Profiles	7905.32	881.82 ***	1
3 Profiles	7240.20	688.89 **	0.92
4 Profiles	7093.35	177.51	0.92

Note. SSA BIC = Sample Size-Adjusted Bayesian Information Criterion, LRT = Lo–Mendell–Rubin Likelihood Ratio Test. ** *p* < 0.01, *** *p* < 0.001.

## Appendix A

**Table A1:** Correlations between Variables.

		2	3	4	5	6	7	8	9	10	11	12	13	14	15
1	CSE	**0.83**	**0.20**	**0.29**	**0.37**	**−0.21**	**−0.15**	0.06	**−0.22**	**0.32**	**0.40**	**0.27**	−0.09	**0.23**	**0.21**
2	CPI	1	**0.34**	**0.15**	0.10	−0.04	0.06	0.01	**−0.13**	**0.20**	**0.29**	**0.19**	−0.01	**0.13**	**0.15**
3	Creat. Act.		1	**0.18**	**0.14**	−0.04	0.04	0.09	−0.07	**0.20**	**0.19**	**0.23**	0.06	0.10	**0.24**
4	Bal. Coh.			1	**0.71**	**−0.55**	**−0.31**	0.07	**−0.23**	**0.74**	**0.53**	**0.28**	−0.05	**0.32**	**0.24**
5	Bal. Flex.				1	**−0.37**	**−0.20**	**0.29**	**−0.22**	**0.64**	**0.46**	**0.28**	**−0.14**	**0.31**	**0.25**
6	Disengag.					1	**0.69**	**0.37**	**0.60**	**−0.51**	**−0.41**	**−0.23**	0.01	**−0.32**	**−0.20**
7	Enmes.						1	**0.48**	**0.58**	**−0.33**	**−0.29**	**−0.14**	0.03	**−0.23**	**−0.12**
8	Rigid							1	**0.24**	0.05	−0.04	−0.03	**−0.15**	−0.04	−0.03
9	Chaotic								1	**−0.36**	**−0.32**	**−0.17**	**0.15**	**−0.25**	**−0.15**
10	Communic.									1	**0.69**	**0.28**	**−0.12**	**0.33**	**0.26**
11	Family sat.										1	**0.26**	−0.05	**0.22**	**0.23**
12	Enc. Nov.											1	**−0.53**	**0.82**	**0.85**
13	Enc. Nonc.												1	**−0.70**	**−0.47**
14	Supp. Persev.													1	**0.76**
15	Enc. Fantas.														1

Note. *N* = 303. All correlations above or equal |0.12| are statistically significant at *p* < 0.05 and are bolded. CSE = creative self-efficacy, CPI = creative iersonal Identity; Creat. Act = creative activity; Bal. Coh. = balanced cohesion; Bal. Flex. = balanced flexibility; Diseng. = disengagement; Enmes. = enmeshed; Rigid. = rigidity; Chaotic = chaos; Communic. = communication; Family. Sat = family satisfaction; Enc. Nov. = encouragement novelty; Enc. Nonc. = encouragement nonconformity; Supp. Persev. = support for perseverance; Enc. Fantas. = encouragement fantasy.
